# Systematic review and meta-analysis of *Campylobacter* species infections in humans and food-producing animals in Nigeria, 2002-2023: The imperative of a One Health control approach

**DOI:** 10.1016/j.onehlt.2025.101029

**Published:** 2025-04-08

**Authors:** Emmanuel O. Njoga, Victory C. Nnaemeka, Ishmael F. Jaja, James W. Oguttu, John A. Nwanta, Kennedy F. Chah

**Affiliations:** a Department of Veterinary Public Health and Preventive Medicine, Faculty of Veterinary Medicine, University of Nigeria, Nsukka 410001, Nigeria; b Department of Microbiology, Faculty of Biological Sciences, University of Nigeria, Nsukka 410001, Nigeria; c Department of Paediatrics, Oxford Vaccine Group, University of Oxford, United Kingdom; d Department of Livestock and Pasture Science, University of Fort Hare, Alice 5700, South Africa; e Department of Agriculture and Animal Health, College of Agriculture & Environmental Sciences, University of South Africa, South Africa; f Department of Veterinary Microbiology and Immunology, Faculty of Veterinary Medicine, University of Nigeria, Nsukka 410001, Nigeria

**Keywords:** *Campylobacter species*, Zoonosis, Prevalence, meta-analysis, meta-regression, Nigeria, One Health

## Abstract

Zoonotic *Campylobacter* species (ZCS), particularly *C. jejuni*, *C. coli,* and *C. lari*, pose significant health risks to humans and food-producing animals (FPAs). This study investigates the prevalence, geospatial and temporal distributions of Campylobacter species infections (CSI) in Nigeria from 2002 to 2023 through a systematic review and meta-analysis of 40 studies, adhering to PRISMA 2020 guidelines. The overall pooled prevalence of CSI was 33 % (95 % CI: 25 % - 41 %), with significant variations among hosts: poultry (42 %, 95 % CI: 27 % - 57 %), humans (30 %, 95 % CI: 23 % - 38 %), and cattle (21 %, 95 % CI: 15 % - 32 %). In humans, the prevalence were 20.3 % in healthy individuals, 23.8 % in diarrheic patients, and 34.2 % in HIV patients. *C. coli* was the predominant isolate in humans (87.5 %) and cattle (38.1 %), while *C. jejuni* was prevalent in poultry (76.2 %). The North-West geopolitical zone exhibited the highest geospatial prevalence at 40 % (95 % CI: 23 % - 57 %). Meta-regression analysis indicated that diagnostic method did not significantly impact prevalence (*p* = 0.2170), but sample type explained 25.70 % of the between-study variance (Wald χ^2^ (2) = 33.10, *p* < 0.0001). Poultry samples showed the highest predicted prevalence at 47.8 % (95 % CI: 39.01 % - 56.51 %), significantly greater than cattle at 18.3 % (95 % CI: 8.9 % - 27.8 %; coefficient = 0.2942, *p* < 0.001). Sensitivity analyses showed minimal changes in pooled prevalence (33 % to 32 %), confirming the robustness of findings despite high heterogeneity (I^2^ = 99.48 % vs. 99.52 %). Temporal analysis indicated that poultry infections peaked between 2016 and 2020. These findings highlight the critical importance of implementing effective biosecurity measures and enhancing food safety practices to mitigate *Campylobacter* transmission in Nigeria, particularly in poultry and the North-West zone, which exhibited the highest prevalence rates. The adoption of One Health control approach, including the “farm to fork” principle, is strongly recommended to limit human *Campylobacter* infections by ensuring comprehensive food safety practices throughout the livestock production and processing value chains.

## Highlights

• Overall prevalence of zoonotic *Campylobacter* infections in Nigeria was 30.6 %, *C. coli & C. jejuni* were the predominant spp.
• The host-specific prevalence were 43.9 %, 23 %, and 18.9 % for poultry, humans, and cattle, respectively
• North-west (43.6 %) and South-west (33.1 %) zones of Nigeria had the top two geospatial *Campylobacter* infections
• *Campylobacter* infection in poultry was highest all through the study period but infections peaked for all the 3 hosts between 2016 and 2020
• Biosecurity and “farm to fork” principle of food safety along livestock production and processing lines are recommended

## 1 Introduction

*Campylobacter* species are leading causes of bacterial foodborne gastroenteritis worldwide, with *C. jejuni* and *C. coli* accounting for 95–98 % of human campylobacteriosis cases [1,2]. These pathogens cause approximately 500 million cases annually, resulting in substantial health and economic burdens [3]. The global economic impact is staggering, with annual losses of 8.6 billion US dollars attributed to human *Campylobacter*-associated illnesses and 12.6 billion US dollars to production losses in both humans and animals [3].

While *C. jejuni* and *C. coli* are the most clinically relevant species [4], other species have also been implicated in human infections. These include *C. lari*, *C. concisus*, *C. rectus*, *C. hyointestinalis*, *C. insulaenigrae*, *C. sputorum*, *C. helveticus*, *C. fetus*, *C. mucosalis*, *C. upsaliensis*, and *C. ureolyticus* [5,6]. Warm-blooded animals, especially food-producing animals (FPAs) like poultry and cattle, are susceptible to *Campylobacter* species infections (CSI) [7,8]. These animals serve as major reservoirs for transmission to humans via the food chain [9]. The prevalence of *Campylobacter* is high in poultry, where birds may be colonized with up to 10^8^ colony-forming units (CFU) per gram of faeces without exhibiting any apparent clinical signs [10].

The thermal preference of *Campylobacter* species, particularly *C. jejuni* and *C. coli*, contributes to their prevalence in these animals. These bacteria grow optimally at 42 °C, which coincides with the body temperature of birds. This thermal adaptation explains their higher incidence in poultry and, by extension, their increased prevalence in tropical and subtropical regions where environmental conditions are more favourable for their survival and transmission.

Zoonotic *Campylobacter* species (ZCS) are primarily transmitted via the faecal-oral route and environmental contamination [11]. Human infections often result from consuming contaminated raw or undercooked animal products, including poultry, beef, unpasteurized milk, and untreated water [12,13,14]. Infected pets and other domesticated animals can also transmit ZCI through direct contact or environmental contamination [3]. Additional transmission routes include exposure to untreated animal manure, contaminated recreational waters, and infected wild bird faeces in public spaces [15]. Reverse zoonotic transmission further complicates *Campylobacter* epidemiology, where humans can infect animals [16].

Coker et al. [17] have shown that children under five years old are disproportionately affected by *Campylobacter* infections. A 2021 systematic review reported that in low- and middle-income countries, the incidence of *Campylobacter*-associated diarrhoea in children fewer than five was 9729 per 100,000 person-years compared to 1340 per 100,000 in children aged five to fifteen years [18]. This heightened vulnerability is attributed to their developing immune systems and increased likelihood of consuming contaminated food or water [19].

Given this context, it is concerning that *Campylobacter* infections are prevalent in Nigerian poultry flocks, with rates as high as 62.7 % reported in some regions [20]. However, there remains a paucity of comprehensive data on the spatial and temporal dynamics of zoonotic *Campylobacter* infections (ZCI) in both humans and food-producing animals (FPAs). Addressing these complexities is crucial, and the One Health concept - emphasizing the interconnectedness of human, animal, and environmental health - plays a vital role in the sustainable management of zoonotic infections like *Campylobacter* [4].

In response to these knowledge gaps, this systematic review and meta-analysis was conducted to synthesize available data on the prevalence and distribution of *Campylobacter* infections in Nigeria. Our findings aim to inform targeted public health interventions and guide national strategies for controlling *Campylobacter* transmission through an integrated One Health approach.

## 2 Materials and methods

### 2.1 Study area

**Fig. 1:**
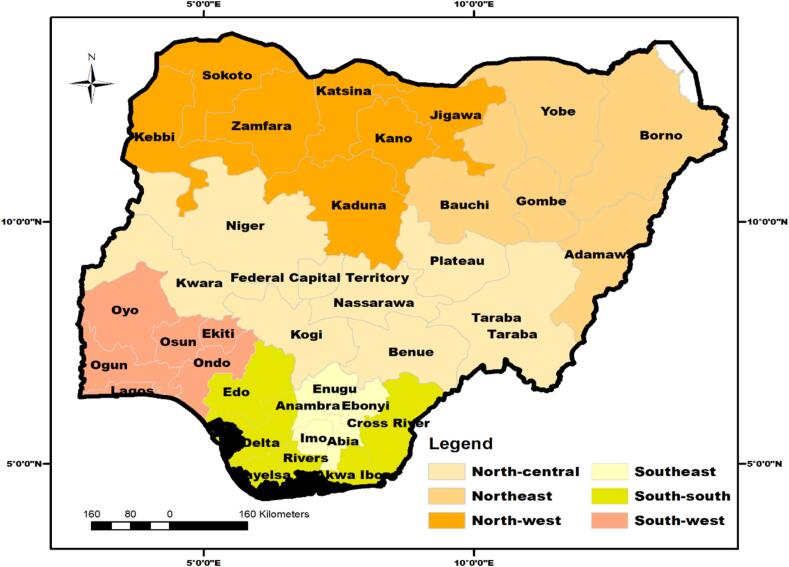
Map of Nigeria showing the 36 states of the federation and the federal capital territory, grouped into six geopolitical zones

Nigeria (Fig. 1) is a West African country in the Gulf of Guinea. Situated between Latitude 9° 04′ 39.9“ North and Longitude 8° 40’ 38.8” East, Nigeria is the 6th most populous country in the world, with an estimated human population of 236.4 million as of April 8, 2025, equivalent to 3 % of the global population [21]. Nigeria's mean annual precipitation is 1165 mm [22]. The southern regions experience heavy rainfall during the rainy or wet season (May to October), usually above 2000 mm, and up to 4000 mm in the Niger Delta region [22]. The ambient temperature ranges from 18 °C to 37 °C and 25 °C to 48 °C during the rainy/cold (winter) and dry/hot (Summer) seasons, respectively [22], depending on the region of the country. These climatic factors seem to help zoonotic pathogens to thrive in Nigeria and other tropical settings [23]. Subsistence and medium-scale production of FPAs reared under extensive and semi-extensive husbandry management systems; and suboptimal meat processing practices, widely practised in rural settings in Nigeria also enhances the spread of zoonotic pathogens [24,25,26,27,28].

### 2.2 Literature search and retrieval

This systematic review and meta-analysis adhered to the PRISMA 2020 guidelines [29]. We searched Scopus, PubMed/Medline, Web of Science, and African Journals Online (AJOL) for peer-reviewed articles published in English between January 1, 2002, and December 31, 2023. The search strategy employed was:

(*Campylobacter* OR campylobacteriosis) AND (Nigeria OR Nigerian) AND (prevalence OR incidence OR occurrence) AND (human OR cattle OR poultry OR chicken OR “food-producing animals”). All references were imported into Zotero reference management software for deduplication and organization. Two authors independently screened titles and abstracts and performed quality assessment with a third author resolving disagreements with respect to the inclusion and exclusion criteria below.

### 2.3 Inclusion and exclusion criteria

**Inclusion Criteria:**

a) Cross-sectional studies reporting prevalence of *Campylobacter* species infections in Nigeria
b) Samples from cattle, edible cattle products, poultry, chicken meat, or humans
c) Peer-reviewed articles published in English
d) Publication date between January 1, 2002, and December 31, 2023
e) Studies with a Modified Downs and Black checklist score of 15 or higher

**Exclusion Criteria:**

a) Studies not conducted in Nigeria
b) Studies not reporting prevalence data
c) Non-peer-reviewed articles, conference abstracts, or gray literature
d) Studies published in languages other than English
e) Studies with a Modified Downs and Black checklist score below 15

### 2.4 Data extraction and preparation

Data from the included studies were extracted into Microsoft Excel by two authors working independently. The extraction captured information on the author, publication year, study location, sample type (human, poultry, cattle), sample size, number of positive cases, and detection methods used. Any discrepancies in data extraction were resolved through discussion or consultation with the third author.

### 2.5 Pooled prevalence estimation

We employed a random-effects model to calculate the pooled prevalence estimate, given the expected heterogeneity among studies. This was implemented using the ‘meta summarize’ command in Stata. The level of heterogeneity was quantified using the I^2^ and τ^2^ statistics, which are automatically calculated and displayed at the base of the forest plot.

### 2.6 Subgroup analyses

We performed subgroup analyses based on sample type and geographical zone (North-Central, North-East, North-West, South-East, South-West) using the ‘meta summarize’ command with the ‘subgroup ()’ option in Stata. The significance of subgroup differences was assessed using the Q test for subgroup heterogeneity (Q_b), rather than overall heterogeneity estimation. This test provides a Q_b statistic and an associated *p*-value. We considered differences between subgroups to be statistically significant if the p-value was less than 0.05. Additionally, we calculated the I^2^ statistic for subgroup differences to quantify the proportion of the variation between subgroups that is due to true subgroup differences rather than chance. An I^2^ value greater than 50 % was considered indicative of substantial true differences between subgroups.

### 2.7 Publication bias assessment

Publication bias was assessed visually using a funnel plot and statistically using Egger's test, implemented through the ‘meta bias’ command. The funnel plot was centred on the overall pooled prevalence estimate.

### 2.8 Additional analyses

#### 2.8.1 Sensitivity analysis

A sensitivity analysis was conducted by removing studies with sample sizes less than 100. This was done to assess the robustness of our findings and to determine if smaller studies disproportionately influenced the overall results. We used the ‘meta summarize’ command in Stata, comparing the pooled prevalence estimates and heterogeneity statistics before and after the removal of these studies.

#### 2.8.2 Meta-regression analyses

Two separate meta-regression analyses were performed using the ‘meta regress’ command in Stata:

*Sample type:* We examined the association between sample type (poultry, human, and cattle) and *Campylobacter* prevalence. This model aimed to quantify the differences in prevalence across these categories.

*Detection method*: We investigated whether the detection method (cultural vs. non-cultural) significantly influenced the reported prevalence estimates.

### 2.9 Statistical analysis

All analyses were conducted using Stata version 18.5 (StataCorp, Timberlake, Oxford, UK). We utilized the ‘meta’ suite of commands for the meta-analysis. All statistical tests were two-sided, with a significance level set at *p* < 0.05. Results are reported as pooled prevalence estimates with 95 % confidence intervals. We reported our findings following the PRISMA guidelines.

## 3 Results

### 3.1 Literature search and inclusion in the study

**Fig. 2:**
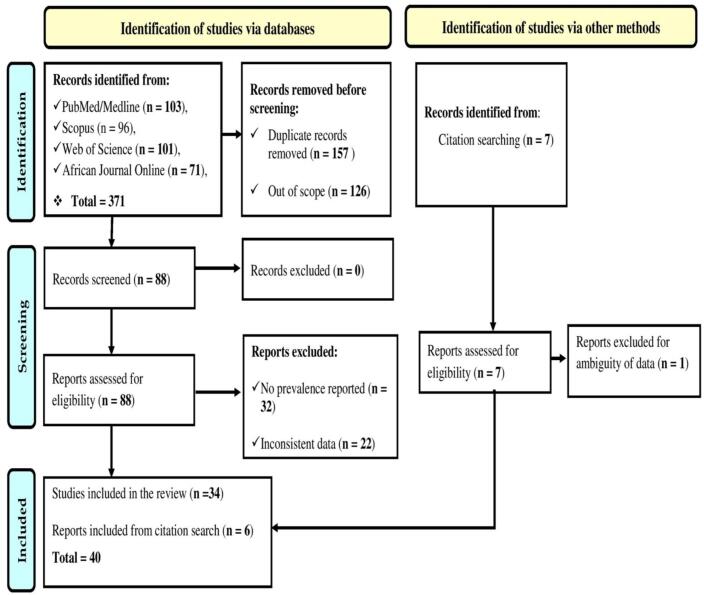
PRISMA flow chart describing the identification, screening, and inclusion criteria for the 40 published papers reviewed in this study.

A total of 371 peer-reviewed papers were found during the literature search. However, only 40 published articles that meet all the eligibility and inclusion criteria were included in the study (Fig. 2). All papers included in the study scored ≥15 (on a 28-point scale) in the Modified Downs and Black checklists [30]. Of the 40 published articles in the study, nine reported the prevalence of CSI in more than one host (species), such that 17, 21 and 13 studies reported CSI in humans, poultry and cattle, respectively.

### 3.2 Study characteristics and data extraction table

Table 1 below summarizes the characteristics of the studies included in the meta-analysis. Each study's author, location, sample type, sample size, frequency of *Campylobacter* occurrence, and detection methods are detailed. This table provides an overview of the data extracted from the studies used to assess the prevalence of *Campylobacter* in various sample types across different geographical zones.

**Table 1:** Data extraction table summarising the characteristics of 40 published studies included in the meta-analysis of *Campylobacter* species infections in cattle, poultry and humans in Nigeria from 2002 to 2023.

S/n	Author	State	Geopolitical zone	Sample type	Sample size	Frequency (%)	Prevalence	Diagnostic methods	*Campylobacter* species reported	JBI
1	[31]	Lagos	South-West	Poultry Faeces	50	15	30	Culture	*C. coli, C. jejuni*	16
2a	[32]	Osun	South-West	Diarrheic children	300	57	19.1	Culture	*C. coli*	18
2b	[32]	Osun	South-West	AH adults	100	6	6	Culture	*C. coli*	18
3	[33]	Oyo	South-West	HIV patients	100	68	68	Culture	*C. upsaliensis, C. jejuni, C. lari, C. coli*	18
4	[34]	Osun	South-West	AH children	602	30	0.5	Culture	*C. coli*	18
5	[35]	Osun	South-West	Diarrheic children	815	357	43.8	Culture	*C. jejuni, C. coli*	18
6a	[36]	Imo	South-East	Raw chicken	200	191	95.5	Culture	*C. jejuni*	17
6b	[36]	Imo	South-East	Raw beef	200	99	49.5	Culture	*Campylobacter spp*	18
7	[37]	Enugu	South-East	Poultry Faeces	275	102	37.1	Culture	*C. jejuni, C. coli, C. lari*	18
8a	[38]	Plateau	North-Central	AH adults	586	53	9	Non-Culture	*C. jejuni, C. coli*	20
8b	[38]	Plateau	North-Central	Poultry Faeces	312	127	40.7	Non-Culture	*C. jejuni, C. coli*	20
8c	[38]	Plateau	North-Central	Cattle Faeces	149	7	4.7	Non-Culture	*C. jejuni, C. coli*	20
9a	[39]	Imo	South-East	Raw chicken	400	101	25.2	Culture	*C. jejuni, C. coli*	17
9b	[39]	Imo	South-East	Raw beef	400	101	25.2	Culture	*C. jejuni, C. coli*	18
10	[40]	Oyo	South-West	AH adults	100	68	68	Culture	*C. jejuni, C. coli, C. lari, C. fetus*	17
11	[41]	Kebbi	North-West	AH adults	150	94	62.7	Culture	*C. coli, C. jejuni, C. lari, C. upsaliensis, C. hyointestinalis*	17
12	[42]	Kaduna	North-West	Diarrheic children	261	40	15.3	Culture	*Campylobacter spp*	19
13	[43]	Ogun	South-West	Poultry Faeces	550	121	22	Non-Culture	*C. coli, Campylobacter spp*	19
14	[44]	Adamawa	North-East	Prepucial fluid	602	99	16.4	Culture	*C. fetus*	20
15	[45]	Borno, Yobe	North-East	Prepucial fluids	270	10	3.7	Culture	*C. fetus*	20
16	[46]	Plateau	North-Central	Poultry Faeces	135	42	31.1	Non-Culture	*C. jejuni, C. coli*	21
17	[47]	Plateau	North-Central	Cattle Faeces	352	65	18.5	Non-Culture	*C. jejuni, C. coli*	21
18a	[48]	Plateau	North-Central	AH adults	300	40	11.3	Non-Culture	*C. jejuni, C. coli*	21
18b	[48]	Plateau	North-Central	Poultry Faeces	360	129	35.8	Non-Culture	*C. jejuni, C. coli*	21
19	[49]	Enugu	South-East	Poultry Faeces	640	124	19.4	Culture	*Campylobacter spp*	20
20	[50]	Enugu	South-East	Poultry Faeces	316	60	18.9	Culture	*Campylobacter spp*	19
21a	[51]	Sokoto	North-West	Pregnant women	57	40	70	Culture	*C. lari, C. coli, C. jejuni*	16
21b	[51]	Sokoto	North-West	Non-pregnant women	23	10	44	Culture	*C. coli, C. jejuni, C. lari*	16
22a	[52]	Sokoto	North-West	AH adults	292	160	54.8	Culture	*C. jejuni, C. coli, C. lari*	17
22b	[52]	Sokoto	North-West	Poultry Faeces	506	152	30	Culture	*C. jejuni, C. coli, C. lari*	17
23	[53]	Enugu	South-East	Raw chicken	204	29	14.2	Non-Culture	*C. jejuni, C. coli*	18
24a	[54]	Imo	South-East	AH adults	988	183	18.5	Culture	*Campylobacter spp*	21
24b	[54]	Imo	South-East	Raw chicken	533	68	12.7	Culture	*C. jejuni*	21
24c	[54]	Imo	South-East	Raw beef	620	140	22.6	Culture	*Campylobacter spp*	16
25	[55]	Benue	North-Central	Poultry Faeces	192	122	63.5	Culture	*C. jejuni*	16
26	[56]	Kaduna	North-West	Processed cow milk	180	29	16.1	Culture	*C. coli, C. jejuni*	18
27	[57]	Kaduna	North-West	HIV patients	230	45	19.6	Culture	*C. jejuni, C. coli, C. fetus, C. hyointestinalis*	18
28	[58]	Lagos	South-West	Poultry Faeces	150	8	5.3	Culture	*C. coli*	18
29	[59]	Enugu	South-East	Diarrheic children	514	43	8.3	Culture	*C. jejuni*	19
30a	[60]	Oyo	South-West	Poultry Faeces	50	5	10	Culture	*C. coli*	15
30b	[60]	Oyo	South-West	Cattle Faeces	200	46	23	Culture	*C. coli*	17
31	Olabode et al., 2017	Abuja	North-Central	Raw beef	75	51	68	Culture	*Campylobacter spp*	15
32	[61]	Oyo	South-West	Raw chicken	252	242	96	Culture	*C. jejuni*	19
33	[62]	Sokoto	North-West	Poultry Faeces	866	672	77.6	Culture	*C. jejuni, C. coli, C. lari, C. hyointestinalis*	19
34	[63]	Sokoto	North-West	Raw chicken	681	558	81.9	Culture	*C. jejuni, C. coli, C. lari*	19
35	[64]	Sokoto	North-West	Cattle Faeces	976	126	12.9	Culture,	*C. jejuni, C. coli, C. lari, C. fetus, C. hyointestinalis*	19
36	[65]	Sokoto	North-West	Raw milk	146	7	4.8	Culture	*C. jejuni, C. coli*	18
37	[66]	Sokoto	North-West	Raw beef	242	54	22.3	Culture	*C. jejuni, C. coli, C. lari*	18
38	[67]	Sokoto	North-West	Poultry Faeces	270	139	51.5	Culture	*C. jejuni, C. coli, C. lari*	19
39	[68]	Kwara	North-Central	Diarrheic adults	306	25	8.2	Culture	*C. jejuni, C. coli*	18
40	[69]	Lagos	South-West	Poultry Faeces	147	108	73.5	Culture	*C. coli, C. jejuni*	17

Cultural methods: Culture, biochemical tests, biotyping; Non-cultural methods: WGS, PCR.

### 3.3 Reported prevalence of *Campylobacter* infections in Nigeria

#### 3.3.1 Overall prevalence of *Campylobacter* from 2002 to 2023

**Fig. 3:**
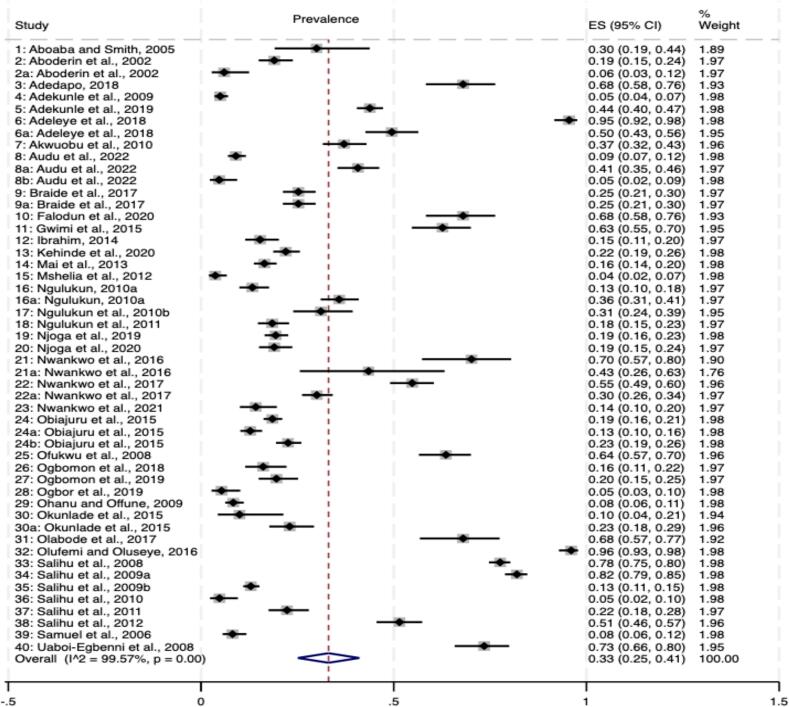
Forest plot of the overall prevalence of *Campylobacter* species infections in humans and food-producing animals in Nigeria from 2002 to 2023. The forest plot (Fig. 3) visualizes the prevalence estimates from individual studies and the overall pooled prevalence. Each horizontal line represents a study, with the square indicating the point estimate and the line showing the 95 % confidence interval. The diamond at the bottom represents the overall pooled prevalence estimate.

Our meta-analysis included 40 studies conducted across Nigeria between 2002 and 2023. The overall pooled prevalence of *Campylobacter* was found to be 33 % (95 % CI: 0.25–0. 44). However, significant subgroup heterogeneity was observed among the studies (I^2^ = 99.48 %, *p* < 0.001), indicating substantial variability in prevalence estimates across different studies (Fig. 3).

### 3.4 Subgroup analyses

#### 3.4.1 Host or species prevalence

**Fig. 4:**
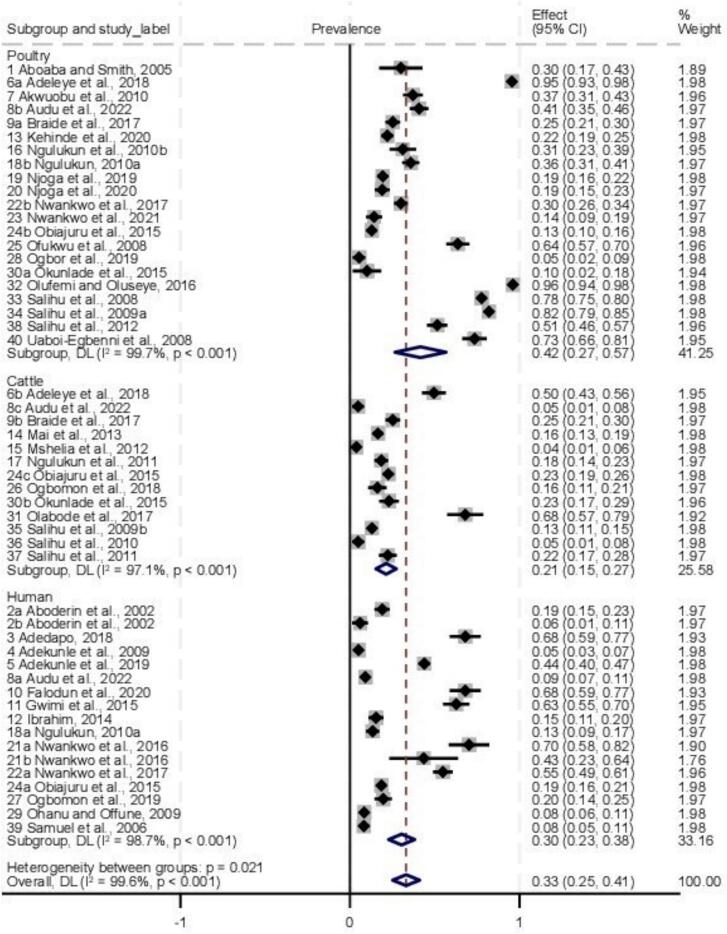
Forest plot of *Campylobacter* prevalence by sample type in Nigeria.

**Fig. 5:**
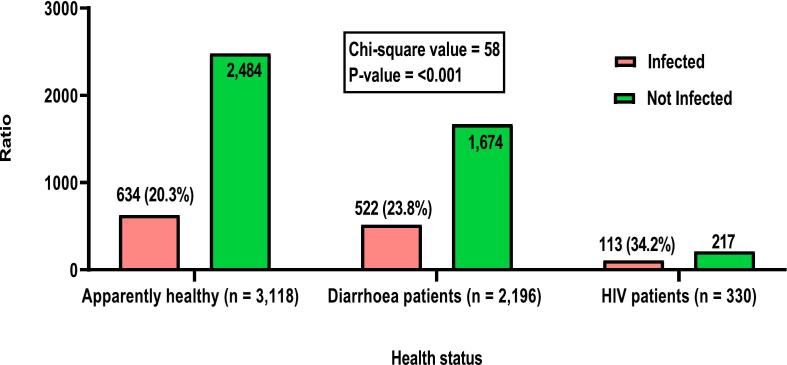
Distribution of human *Campylobacter* infections in Nigeria from 2002 to 2023 according to health status.

The prevalence varied among various hosts, with poultry recording the highest prevalence at 42 % (95 % CI: 0.27–0.57), followed by human samples at 30 % (95 % CI: 0. 23–0. 38) and cattle samples at 21 % (95 % CI: 0.15–0.27) (Fig. 4). Interestingly, the test for subgroup differences showed statistically significant heterogeneity in CSI between the three hosts studied (I^2^ = 99.6 %, p < 0.001), with the infection being significantly associated with poultry (*p* = 0.027) than cattle and humans (Fig. 4). Based on the health status, the prevalence of *Campylobacter* pathogens in humans were 20.3 %, 23.8 % and 34.2 % in apparently healthy individuals, diarrheic patients and HIV patients, respectively (Fig. 3). There was a significant association (*p* = 0.018) between occurrence of *Campylobacter* pathogens and human health status (Fig. 5).

The forest plot visualizes the prevalence estimates for each sample type along with their corresponding 95 % confidence intervals. Each horizontal line represents a sample type, with the square indicating the point estimate and the line showing the 95 % confidence interval. The diamond at the end of each sample type group represents the pooled prevalence estimate for that specific sample types.

### 3.5 *Campylobacter* species detected

**Fig. 6:**
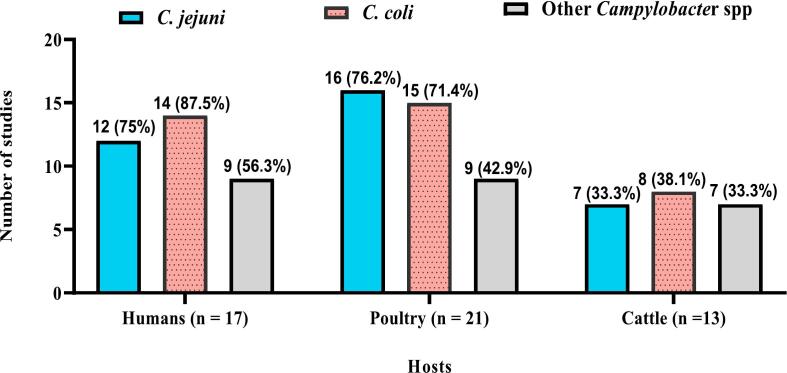
Distribution of *Campylobacter* species isolated from humans, poultry and cattle in Nigeria. Other *Campylobacter* spp. = *C. lari, C. hyointestinalis, C. fetus* and *C. upsaliensis.*

The *Campylobacter* species isolated from human and FPAs samples in Nigeria include *C. jejuni, C. coli*, *C. lari, C. hyointestinalis, C. fetus* and *C. upsaliensis.* Of the 51 studies included in this review, 35 isolated *C. jejuni* (68.6 %) while 37 reported the occurrence of *C. coli* (72.5 %). Other species (*C. lari, C. hyointestinalis, C. fetus* and *C. upsaliensis*) accounted for 49 % prevalence. The *C. coli* was the principal CSI in humans (87.5 %) and cattle (38.1 %) while *C. jejuni* predominated in poultry (76.2 %). Detailed results on the distributions and proportions of *Campylobacter* species detected in humans, poultry and cattle are presented in Fig. 6.

### 3.6 Prevalence by Geographical Location

**Fig. 7:**
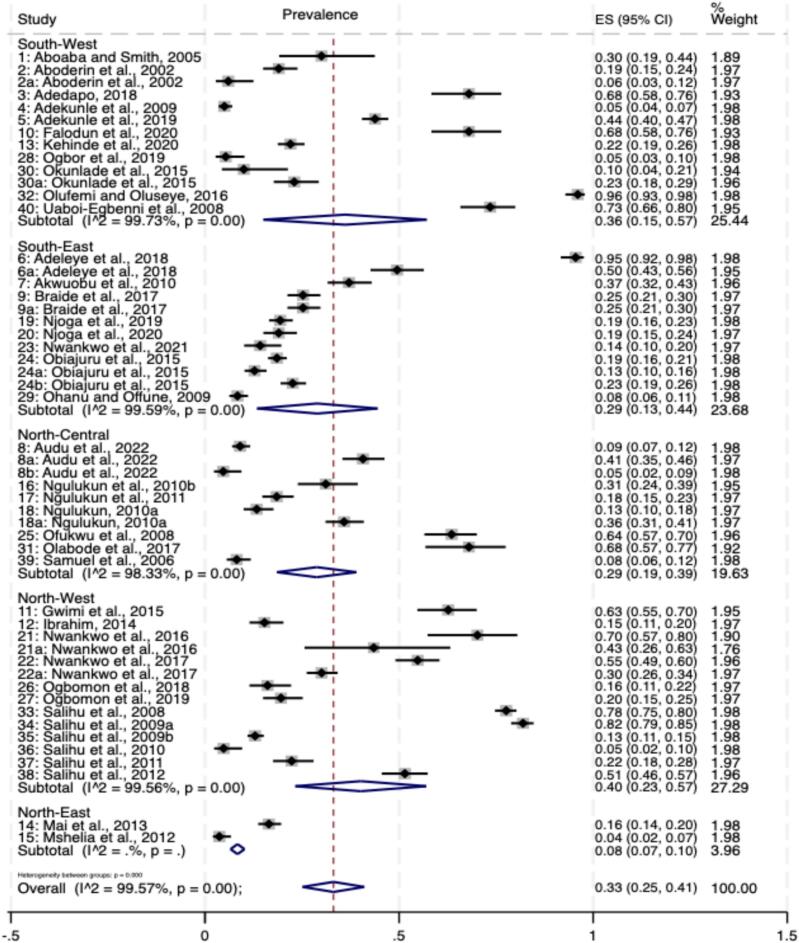
The forest plot visualizes the prevalence estimates by geographical location, with each horizontal line representing a specific geographical zone. The square indicates the point estimate, and the line shows the 95 % confidence interval for each zone. The diamond at the end of each geographical zone group represents the pooled prevalence estimate for that specific location.

Significant differences were also observed across geographical zones (Q_b = 11.77, *p* = 0.019). The North-west (40 %, 95 % CI: 2.3–5.7) and South-West zone (36 %, 95 % CI: 0.15–5.7 had the highest prevalence at 38.7 % (95 % CI: 28.9 % - 48.5 %), while the Northeast zone had the lowest at 8 % (95 % CI: 0.07–0.1) (Fig. 7).

### 3.7 Temporal distribution of *Campylobacter* infections in Humans, poultry and cattle in Nigeria

**Fig. 8:**
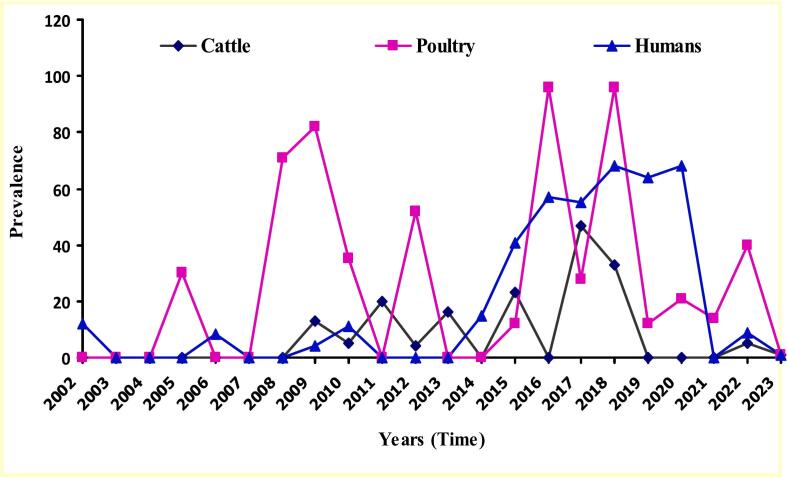
Temporal distributions of documented *Campylobacter* species infections in cattle, poultry and humans in Nigeria from 2002 to 2023

The trends and peaks of documented CSI in humans, poultry and cattle in Nigeria from 2002 to 2023 are presented in Fig. 8. Temporal distribution of CSI in the three hosts showed that the infection was highest in poultry from 2005 to 2022, followed by humans and least in cattle (Fig. 6). The poultry infection was not reported between 2002 and 2004. Thereafter, the infection rose in 2005, fluctuated between 2005 and 2022, but peaked in 2009, 2016 and 2018. Afterwards, CSI in poultry dwindled to basal level in 2023. Human CSI remained mainly at the basal level between 2002 and 2013. Later, it rose steadily and peaked between 2018 and 2020, declining to zero in 2021. The bovine infection was low (about 20 % prevalence) until 2017, when it peaked at 56 % before dropping to zero in 2019 (Fig. 6). Overall, poultry harboured the highest prevalence of ZCI from 2005 to 2018, but the infection peaked in all three hosts studied between 2016 and 2020.

### 3.8 Publication bias

**Fig. 9:**
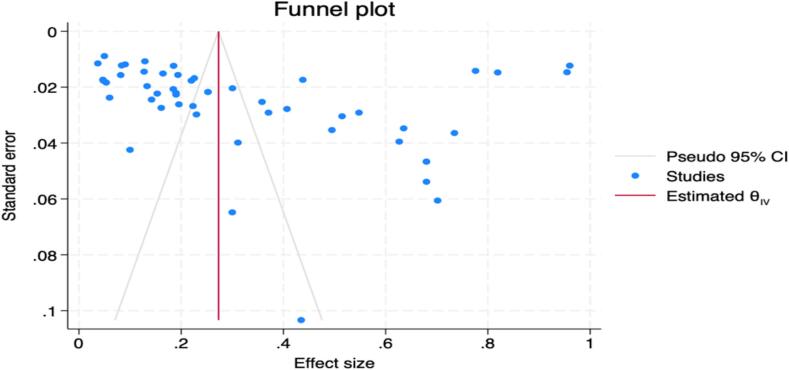
Funnel plot of *Campylobacter* prevalence studies in Nigeria. The x-axis shows prevalence estimates, and the y-axis represents standard error. Each dot represents an individual study, with the vertical line indicating the overall pooled prevalence estimate and diagonal lines representing 95 % confidence limits. Asymmetry may suggest publication bias.

Egger's test indicated significant small-study effects (z = 2.35, *p* = 0.0189), suggesting potential publication bias. The funnel plot (Fig. 9) showed most effect size estimates clustered at the top, indicating larger sample sizes or greater precision. While the distribution appeared somewhat symmetrical, several studies were scattered outside the funnel's boundaries, suggesting heterogeneity or potential outliers. The central vertical line, representing the overall pooled prevalence estimate at 0.30, aligned with our meta-analysis

### 3.9 Meta-regression analysis

We conducted meta-regression analyses to explore potential sources of heterogeneity in *Campylobacter* prevalence estimates. Two separate models were examined: one based on sample source and another on detection method.

*Sample Type*: Meta-regression by sample type (poultry, human, and cattle) revealed significant differences in *Campylobacter* prevalence (Wald χ^2^ (2) = 33.10, *p* < 0.0001). The model explained 25.70 % of the between-study variance. Poultry samples showed a significantly higher prevalence compared to cattle (coefficient = 0.2942, *p* < 0.001), while the difference between human and cattle samples was not statistically significant (coefficient = 0.0542, *p* = 0.370).

*Detection Method*: The meta-regression by detection method (cultural vs. non-cultural) did not show a significant difference in prevalence estimates (Wald χ^2^(1) = 1.52, *p* = 0.2170). The model explained only 1.16 % of the between-study variance, with non-cultural methods showing a non-significant trend towards lower prevalence estimates (coefficient = −0.0946, p = 0.217). Table 2 presents the detailed results of both meta-regression analyses – host species or sample type and detection method.

**Table 2:** Meta-regression results for host species or sample type and detection method for *Campylobacter* species infections in humans and food-producing animals in Nigeria.

Variable	Coefficient	SE	P-value	Predicted Prevalence (95 % CI)
Host species or sample type				
Poultry	0.2942	0.0598	<0.001	47.76 % (39.01 % - 56.51 %)
Human	0.0542	0.0604	0.37	23.75 % (15.10 % - 32.40 %)
Cattle (ref)	–	–	–	18.34 % (8.87 % - 27.81 %)
Detection Method				
Non-cultural	−0.0946	0.0766	0.217	26.74 % (17.37 % - 36.11 %)
Cultural (ref)	–	–	–	36.20 % (27.54 % - 44.85 %)

Model Statistics:

Sample Type: *N* = 94, R^2^ = 25.70 %, Residual I^2^ = 99.21 %, Wald χ^2^ (2) = 33.10, *p* < 0.0001.

Detection Method: *N* = 51, R^2^ = 1.16 %, Residual I^2^ = 99.45 %, Wald χ^2^ (1) = 1.52, p = 0.2170

### 3.10 Sensitivity analysis

A sensitivity analysis was conducted by excluding studies with sample sizes less than 100 to address potential small-study effects identified by Egger's test (z = 2.35, *p* = 0.0189). This analysis retained 46 of the original 51 studies. The pooled prevalence estimate decreased slightly from 33 % (95 % CI: 2.5–4.1) in the full dataset to 32 % (95 % CI: 2.4–3.9) after excluding smaller studies (Table 1*S*). Heterogeneity remained high in both analyses (full dataset: I^2^ = 99.47 %; reduced dataset: I^2^ = 99.52 %) (Figure1*S*). The change in τ^2^ was minimal (from 0.0664 to 0.007) between the full and reduced datasets, respectively.

## 4 Discussion

Our meta-analysis of CSI in cattle, humans, and poultry in Nigeria from 2002 to 2023 which shows an overall prevalence of 33 % is high and very significant from public health, food safety and One Health perspectives. The significance is predicated on the fact that FPAs, especially poultry and cattle, are the major reservoirs of *Campylobacter* infection for onward transmission to humans, principally through the food chain [20]. The overall prevalence of 33 % is higher than 10.2 % reported in humans and FPAs from Ethiopia [70]. The discrepancy in the findings could be due to sample size (only 12 studies met the inclusion criteria for meta-analysis in the Ethiopian study), *Campylobacter* detection method and the researcher's diagnostic competencies.

The subgroup analysis in which poultry samples exhibited the highest prevalence at 42 % revealed significant variations in prevalence across different hosts. These findings align with global trends where poultry is often identified as the primary reservoir for *Campylobacter* species. The high prevalence in poultry could be attributed to the similarity between the physiological temperature of poultry (42 ± 1 °C) and the optimal growth temperature of ZCS (42 ± 1 °C). This situation is particularly concerning given that chicken and beef are Nigeria's most consumed meat types [71,72], posing a substantial risk for human campylobacteriosis through the food chain. This calls for stringent farm and slaughterhouse hygiene to reduce the colonisation of FPAs and contamination of processed meats with ZCS, to reduce the odds of human CSI and the associated food safety and public health problems.

The prevalence of *Campylobacter* infection in humans at 30 % is lower than the rates reported in Egypt (48 %), Pakistan (54.6 %), and China (85.7 %) [73,74,75]. For poultry, the reported prevalence of 42 % is higher than that of Iran (25.1 %) and Peru (36.2 %) but lower than that of Ethiopia (70 %) [76,77,78]. In cattle, the prevalence of 21 % is higher than 13.5 % in Ethiopia but lower than 30.9 % in Bangladesh and 33.3 % in Malaysia [79,80,81]. These disparities in findings may be attributed to variations in diagnostic methods, researchers' diagnostic proficiency, farm-level biosecurity and hygiene practices, hosts' health and physiological status, and other epidemiological factors influencing infection dynamics in different study locations. Given the dependency of reducing human *Campylobacter* infection on controlling animal infection [82], it is essential to implement improved biosecurity and hygiene measures across livestock production and processing value chains to minimize *Campylobacter* infection in FPAs and reduce the risk of human infection through occupational exposure and consumption of contaminated animal products.

Geographical analysis revealed significant differences across zones (Qb = 11.77, *p* = 0.019), with the top two prevalence rates recorded in the North-West and South-West geopolitical zones. This regional variation could be attributed to differences in climate, animal husbandry practices, and food preparation habits. The preponderance of CSI in the two geopolitical zones calls for concerns because the zones are the most populated in Nigeria and the possibility of rapid disease spread in densely populated areas could result in a nationwide outbreak of human campylobacteriosis.

The finding that *C. jejuni* and *C. coli,* the two most pathogenic species responsible for more than 95 % of the global cases of campylobacteriosis [4,1], were the predominant *Campylobacter* species found in humans and FPAs in this study portends significant public health and food safety concerns. The significance borders on the high infectivity of these highly zoonotic pathogens, such that just about 40 colony-forming units (CFU) of the organisms produced diseases in susceptible human and animal hosts [83,84]. However, Janssen et al. [84] reported that the infective dose can be as low as 100 cells in immune-compromised patients, and ingestion of 500–800 viable cells of *C. jejuni* resulted in campylobacteriosis in an apparently health individual. The significance is also predicated on the fact that ZCS, particularly *C. jejuni,* are major causes of abortion, infertility and other reproductive problems in FPAs, particularly small ruminants and cattle [85]. This could lead to a diminution of edible animal products (meat and milk), which is a public health problem that could worsen the already precarious animal protein scarcity in most developing countries like Nigeria.

Although there is no consistent pattern on the temporal distribution of CSI in the three hosts studied from 2002 to 2023, *Campylobacter* infection in poultry was highest for most parts of this period. The infections peaked in all the host species between 2016 and 2020. This could be due to the effect of climate change, including rising temperatures (due to global warming) and flooding, which favour the proliferation and transmission of food and waterborne diseases, especially campylobacteriosis in tropical settings [86,87]. Flooding could aid the transmission of CSI through the contamination (with animal dung) of fruit and vegetable gardens and natural water bodies, which may be the only available source of drinking water for both humans and animals in rural areas. Unfortunately, Nigeria has experienced the worst flooding in its national history within the last decade due to hefty rainfall and the release of excessive amounts of water from the Lagdo Dam in the neighbouring country of Cameroon [88]. The temporal pattern of CSI documented in this study underscores the role of planetary health in the epidemiology of infectious diseases. It accentuates the need for a coordinated One Health approach to control climate change, which appears to drive CSI in both human and animal populations.

Our meta-regression analysis provided crucial insights into the sources of heterogeneity in *Campylobacter* prevalence. Sample type emerged as a significant factor (Wald χ^2^ (2) = 33.10, *p* < 0.0001), explaining 25.7 % of the between-study variance. Poultry samples showed a significantly higher prevalence compared to cattle (coefficient = 0.2942, *p* < 0.001), reinforcing the role of poultry as a major reservoir. Interestingly, the detection method (cultural vs. non-cultural) did not significantly influence prevalence estimates (Wald χ^2^ (1) = 1.52, *p* = 0.2170), suggesting robustness across different diagnostic approaches.

Given the high heterogeneity observed in our meta-regression analysis and the lack of a clear linear temporal pattern, we opted to present these trends using line graphs rather than forest plots. This approach allowed us to better visualize the complex, non-linear dynamics of *Campylobacter* prevalence over time, highlighting the potential influence of various environmental and socio-economic factors that may not be captured by simple linear trends. The temporal analysis from 2002 to 2023 revealed fluctuating trends, with peaks observed between 2016 and 2020 across all host species. This pattern could be linked to climate change effects, including rising temperatures and increased flooding events in Nigeria, which may favour the proliferation and transmission of *Campylobacter* [86,87]

Our sensitivity analysis, excluding studies with sample sizes less than 100, showed a slight decrease in pooled prevalence from 33 % to 32 %, with persistently high heterogeneity. This suggests that our findings are robust and not significantly influenced by small-sample size effects.

## 5 Limitations

While our meta-analysis provides valuable insights into *Campylobacter* prevalence in Nigeria, few limitations may exist. The study included studies that did not use PCR to confirm Campylobacter infection status, The study protocol was not pre-registered in databases such as PROSPERO (International Prospective Register of Systematic Reviews). The high heterogeneity observed across studies suggests that factors beyond those examined in our meta-regression may influence *Campylobacter* prevalence. We also acknowledge that while our meta-regression focused on sample type and detection method due to their direct relevance to how Campylobacter prevalence is assessed across studies, other potential moderators, such as study year and geographic location, were excluded due to inconsistent reporting. Many studies did not specify the exact sampling period, limiting their interpretability in meta-regression. Although there were variation in the quality and uneven distribution (across different regions) of papers included in this study, our sensitivity analysis suggests that these effects were minimal. Despite these limitations, our study provides a comprehensive overview of *Campylobacter* prevalence in Nigeria and offers valuable directions for future research and interventions.

## 6 Conclusion and recommendation

Our meta-analysis reveals a high *Campylobacter* prevalence in Nigeria, with poultry as the primary reservoir. Significant variations across sample types and geographical zones, particularly in the North-West, highlight the need for targeted interventions. Temporal fluctuations, peaking between 2016 and 2020, suggest environmental influences on transmission dynamics. We recommend implementing enhanced biosecurity in poultry production, developing region-specific control strategies, improving slaughterhouse hygiene, and establishing a robust surveillance system that accounts for observed variations. These tailored approaches are crucial to address the complex patterns of *Campylobacter* prevalence observed across Nigeria. Future meta-analyses should capture risk factors associated with *Campylobacter* prevalence and examine how moderating factors like seasonal variations impact prevalence rates. This comprehensive approach will provide a more nuanced understanding of *Campylobacter* epidemiology in Nigeria, enabling more effective control strategies.

## CRediT authorship contribution statement

**Emmanuel O. Njoga:** Writing – review & editing, Writing – original draft, Visualization, Resources, Methodology, Investigation, Formal analysis, Data curation, Conceptualization. **Victory C. Nnaemeka:** Validation, Resources, Methodology, Investigation, Formal analysis. **Ishmael F. Jaja:** Writing – review & editing, Investigation, Funding acquisition. **James W. Oguttu:** Writing – original draft, Validation, Supervision, Project administration, Methodology, Investigation, Formal analysis, Data curation. **John A. Nwanta:** Writing – review & editing, Supervision, Methodology, Investigation. **Kennedy F. Chah:** Writing – review & editing, Supervision, Methodology, Investigation.

## Declaration of competing interest

The authors declare that they have no conflict of interest.

## Data Availability

Data will be made available on request.

## Appendix A Supplementary data

Supplementary material Image 1
