# Supplementary material for: Systematic review and meta-analysis of Campylobacter species infections in humans and food-producing animals in Nigeria, 2002-2023: The imperative of a One Health control approach
Source: One Health. 2025 Apr 8;20:101029. doi: 10.1016/j.onehlt.2025.101029 (PMC12017976; doi:10.1016/j.onehlt.2025.101029)
Supplement: Supplementary file 1 — Supplementary material [file mmc1.docx]

**Appendix**

Supplementary Table 1: Sensitivity Analysis Results - Comparison of Full Dataset and Large Studies (n ≥ 100)

| **Analysis** | **Number of Studies** | **Pooled Prevalence (95% CI)** | **I² (%)** | **τ²** |
| --- | --- | --- | --- | --- |
| All studies | 51 | 33% (25% - 41%) | 99.48 | .0664 |
| Studies n ≥ 100 | 46 | 32% (24% - 39%) | 99.52 | 0.07 |

 Supplementary Figure 1: Forest plot of Campylobacter species prevalence in studies with sample size > 100
